# ﻿Checklist, distribution, diversity, and rarity of mayflies (Ephemeroptera) in Slovakia

**DOI:** 10.3897/zookeys.1183.109819

**Published:** 2023-10-27

**Authors:** Patrik Macko, Tomáš Derka, Michaela Šamulková, Milan Novikmec, Marek Svitok

**Affiliations:** 1 Department of Ecology, Faculty of Natural Sciences, Comenius University in Bratislava, Ilkovičova 6, 842 15 Bratislava 4, Slovakia Comenius University in Bratislava Bratislava Slovakia; 2 Department of Biology and General Ecology, Faculty of Ecology and Environmental Sciences, Technical University in Zvolen, Ul. T. G. Masaryka 24, 960 01 Zvolen, Slovakia Technical University in Zvolen Zvolen Slovakia; 3 Department of Ecosystem Biology, Faculty of Science, University of South Bohemia, Branišovská 1760, 30 05, České Budějovice, Czech Republic University of South Bohemia České Budějovice Czech Republic

**Keywords:** Freshwater bioindicators, lowland rivers, rare taxa, species frequency, species richness

## Abstract

Despite the essential role of mayflies (Ephemeroptera) in freshwater ecosystems and their long-term use in research and routine biomonitoring in the Carpathian and Pannonian ecoregions, their distribution data are fragmentary and outdated. All published and unpublished data on mayflies from Slovakia was gathered and a database of > 15,000 species records from 2206 localities built with the aims (i) to critically revise available data and assess the completeness of the species inventory, (ii) to identify hotspots of species diversity, and (iii) to provide a benchmark for assessment of species rarity and conservation status in the region. After the critical revision of the data covering more than 100 years, the occurrence of 109 mayfly species in Slovakia was confirmed. The species inventory appears to be nearly complete, as evidenced by the rarefaction curve and a nonparametric species richness estimator. The highest mayfly gamma diversity was recorded below 500 m a.s.l. and in streams of the fifth order, which can be considered hotspots of mayfly diversity in the region. Six species were last recorded before 1990 and thus can be considered extinct in Slovakia. Twenty-nine species could be classified as very rare, with their occurrence frequency decreasing with increasing altitude and most of them being restricted to large lowland rivers and stagnant water habitats in their floodplains. In conclusion, our study provides comprehensive data on key freshwater bioindicators and suggests increasing conservation priorities, especially in lowland river floodplains occupied by several very rare mayfly species.

## ﻿Introduction

Mayflies (Ephemeroptera) represent one of the oldest insect orders, whose origin dates back to the late Carboniferous (Sartori and Brittain 2015), and together with dragonflies and damselflies (Odonata), they are considered the sister lineage to all other winged orders of insects (Thomas et al. 2013). Their long evolutionary history has resulted in extraordinary morphological and ecological diversity, especially in nymphs, reflected in the diverse types of feeding, locomotion, occupied microhabitats, life cycles or overall requirements for abiotic and biotic environmental conditions (Sartori and Brittain 2015; Jacobus et al. 2019). Mayflies colonise a broad spectrum of freshwater habitats on almost all continents except Antarctica and some remote islands (Sartori and Brittain 2015). However, among the eight major aquatic insect lineages, mayflies are among the least species rich (Dijkstra et al. 2014), but their nymphs constitute a significant part of the macroinvertebrate biomass and production in lotic habitats (Brittain and Sartori 2009). Mayfly nymphs are essential consumers of periphyton and detritus and serve as prey for predators such as fish, amphibians, or predaceous invertebrates (Wallace and Webster 1996; Baptista et al. 2006). They participate in maintaining important ecosystem functions, such as bioturbation, bio-irrigation, decomposition, and self-cleaning processes, which support the natural balance of the whole system (Jacobus et al. 2019). The cosmopolitan distribution of mayflies and their high-water quality requirements make them essential bioindicators of water and habitat quality (Baptista et al. 2001; Medina and Vallania 2001; Arimoro and Muller 2010) and an integral part of biomonitoring protocols (Menetrey et al. 2008; Makovinská et al. 2015) and metrics (e.g., EPT richness; Lewin et al. 2013; Wright and Ryan 2016).

The extant global Ephemeroptera fauna encompasses almost 3800 species in 478 genera and 42 families (Sartori and Brittain 2015; Jacobus et al. 2021), with the highest generic diversity occurring in the Neotropics and the lowest in the Palearctic (but with the highest number of recorded species, Barber-James et al. 2008). From a taxonomic perspective, North America and Europe are the best-explored parts of the world (Sartori and Brittain 2015). The last checklist of mayfly fauna covering most of the Western Palearctic reported 369 species in 48 genera and 19 families (Bauernfeind and Soldán 2012). In general, the diversity of mayflies is low in alpine habitats, while meta- and hyporhithral sections in the colline or lower mountain zones are inhabited by many species (Landolt and Sartori 2001; Bauernfeind and Soldán 2012). Some studies also indicate a high diversity of mayflies in pristine lowland rivers (Bauernfeind and Moog 2000), which are currently known to be the most affected by the reduction of potamal specialists (Zedková et al. 2015). However, more extensive data summarising the diversity of mayflies depending on basic characteristics such as hypsometric distribution, stream order or the habitat classification of running waters (according to Illies 1953 and Illies and Botosaneanu 1963) are largely absent in many European regions, and the current knowledge is thus based on only a few studies (e.g., Landa and Soldán 1989; Bauernfeind and Moog 2000; Landolt and Sartori 2001).

The first faunistic records of mayflies from today’s territory of Slovakia extended to the Austro-Hungarian Empire (Mocsáry 1875, 1878; Petricskó 1892), and the first “checklist” was presented by Mocsáry (1899), who reported 11 species. This list was later expanded to 33 species (Pongrácz 1914). Later, most of the research on mayflies was local and faunistic in nature, often associated with the first records of several species (for a synopsis, see Derka 2006). Previous knowledge was summarised by Landa (1969), who processed data on the distribution, ecology, and taxonomy of 78 mayfly species reported from Czechoslovakia, 68 of which were recorded in today’s territory of Slovakia. Subsequent decades have seen significant progress in mayfly taxonomic knowledge in Central Europe, with revisions of several groups and descriptions of many new species (e.g., Landa 1970; Sowa 1971; Sowa 1981; Sowa and Soldán 1986; Klonowska et al. 1987). Extensive faunistic-ecological investigations consequently characterised the turn of the 1970s and 1980s, and new data on the distribution and ecology of mayflies were obtained as part of broadly focused hydrobiological studies in Slovakia (for a synopsis, see Derka 2006). Landa and Soldán (1989) summarised the long-term research on mayflies in Czechoslovakia concerning water quality, and the number of species reported from Slovakia rose to 94. Finally, the latest catalogue contains as many as 123 species from 37 genera and 16 families, unfortunately without a closer specification of the respective species findings (Derka 2003a) but with a relatively detailed overview of their autecological characteristics (Derka 2003b).

Despite the long tradition of European mayfly faunistic research, checklists are missing in several European countries, including Slovakia, and only a few of them can be considered reliable [e.g., Czech Republic – Zahrádková et al. (2009); Germany, Austria, and Switzerland – Haybach (2010); Austria – Weichselbaumer et al. (2015); Serbia – Petrović et al. (2015); Kosovo – Xërxa et al. (2019); Croatia – Vilenica et al. (2021)]. Our first aim was (i) to critically revise the species list and assess the completeness of the species inventory in Slovakia. Hydro-morphological modifications of rivers, construction of hydropower plants, water pollution, the spread of invasive species and the effects of climate change have recently caused excessive degradation and loss of natural freshwater habitats (Dudgeon et al. 2006; Carpenter et al. 2011), leading to a significant change in communities and a decrease in freshwater biodiversity (Zedková et al. 2015; Sánchez-Bayo and Wyckhuys 2019; Rumschlag et al. 2023). Therefore, we also aimed (ii) to identify regional hotspots of mayfly species diversity and (iii) to provide a benchmark for assessing species rarity and conservation status in the region.

## ﻿Materials and methods

### ﻿Study area

Although the majority of the area of Slovakia (49,035 km^2^; 16°50–22°34'E, 47°44–49°37'N) belongs to the Carpathian Mountains system (Mráz and Ronikier 2016), the territory belongs to the Carpathian and Pannonian ecoregions (Illies 1978; Hók et al. 2019). Substantial landscape diversity (from lowlands to mountains) results in high variability of annual temperature (~ 0.3–11.4 °C), precipitation (500–1400 mm) and elevation (94–2654 m a.s.l.). Most of the largest rivers originate in the central arch of the Western Carpathians and drain to the Danube River basin. Up to 47,056 km^2^ of the country belongs to the Black Sea drainage area, whereas the minority (1953 km^2^) drains into the Baltic Sea (Vistula River Basin; Miklós 2002).

### ﻿Dataset

The data in this study are compiled from two primary sources:

1. Published data. Records of mayflies in Slovakia were gathered from 91 publications and six monographic studies covering the period from 1905 to 2021 (see Suppl. material 1 for publication references). Only one work was published in 1905, 66 between 1950 and 1999, and 33 since 2000.
2. Field survey. Qualitative and quantitative sampling of mayflies was performed at 317 localities during 2003–2021, with more than 1/3 occurring during 2019–2021. Various lotic and lentic habitats were sampled, from springs to potamal sections of the largest rivers and from the lowlands to the high alpine lakes and ponds. Most of the material processed was represented by nymphs, mainly sampled by the kick netting (Frost et al. 1971) or resulting from individual collections from specific types of microhabitats (e.g., clay banks). A negligible part of the material was represented by adults obtained by sweep netting. The material was preserved in situ with 96% ethanol or 4% formaldehyde. Subsequently, individuals were examined under a stereomicroscope for assignment to higher taxonomic groups. Finally, most of the individuals were identified to the species level using the identification keys of Bauernfeind and Humpesch (2001), Eiseler (2005) and Krno and Derka (2011). Voucher material is stored in the collection of the Department of Ecology, Comenius University, Bratislava, Slovakia, and the Department of Biology and General Ecology, Technical University, Zvolen, Slovakia.

### ﻿Data handling and analysis

**Figure 1.**
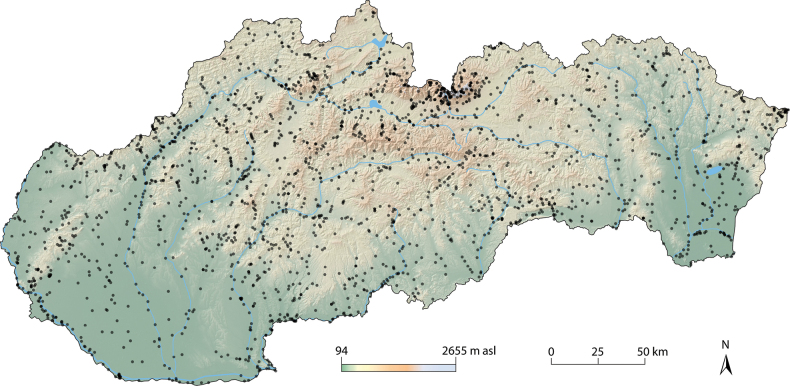
Spatial distribution of localities sampled for mayflies in Slovakia from 1905 to 2021.

Overall, we processed data on mayfly occurrence from 2206 localities (Fig. 1, see Suppl. material 1 for details on collection data) located within the altitudinal range 94–2091 m a.s.l., with a majority situated between 192 and 587 m a.s.l. Lotic ecosystems represented more than 93% of all localities, covering a broad spectrum of aquatic environments from springs and small creeks to large lowland rivers. Alpine lakes and ponds, reservoirs, gravel pits, wetlands or temporary ponds in inundated areas represented lentic ecosystems.

For taxonomic consistency, we used the nomenclature according to Bauernfeind and Soldán (2012), even though several subgenera listed in this study are now commonly considered as genera (e.g., Waltz et al. 1994; Cruz et al. 2021). The final dataset includes only records identified at the species level, except for five taxa with insufficient taxonomical characters in the nymphal stage (refer to Results). Taxa classified by Bauernfeind and Soldán (2012) as “*species inquirenda*” in the latest checklist of mayflies in Europe were excluded from the dataset. This category includes species whose taxonomic position is unclear, and it is impossible to decide whether they represent valid species or should be regarded as subspecies or conspecifics of another species. Another category of excluded taxa includes dubious species, whose findings in Slovakia were based on misidentification as determined in our morphological revision or literature review, demonstrating their highly improbable distribution in the territory (Bauernfeind and Soldán 2012). To evaluate the last record of the species in our territory, we used four time periods (≤ 1990, 1991–2000, 2001–2010, and 2011–2021), which correspond to the period when the collection of the species was published, since in several publications it is not possible to determine the exact time of collection, or it is a longer period.

Geographical coordinates of sampling localities were directly measured in the field, extracted from publications, or determined by the most accurate estimate using the online software Mapy.cz (https://mapy.cz) based on the description of the investigated site in the source publication. Elevation data were determined using Google Earth Pro 7.3.6.9345 or extracted from publications. Repeated samples from the same localities were pooled, and species data were presented only once.

We constructed an analytical sample-based rarefaction curve with unconditional confidence intervals (Colwell et al. 2004) to assess the completeness of the mayfly species inventory. In addition, we used the asymptotic richness estimator Chao2 (Chao 1987) to estimate the total number of mayfly species, including unobserved species. Since the detection probabilities of the species were relatively homogeneous (coefficient of variation of infrequent species = 0.35), we used the bias-corrected form of the estimator (Chao 2005).

To explore the species hypsometric distribution, we created a series of boxplots showing altitudinal optima (median) and variation in species occurrence along a 2000 m elevation gradient. The same approach has been employed to investigate species distributions based on rarity level. To evaluate species rarity based on the frequency of occurrence (i.e., the number of localities with the positive occurrence of the species), we used a five-degree scale developed by Sartori and Landolt (1999): F1 – very rare (up to 10 localities of occurrence), F2 – rare (11–25 localities), F3 – uncommon (26–50 localities), F4 – common (51–100 localities), and F5 – widespread (over 100 localities). This scale was designed for mayflies concerning the order’s chorological specificity and has already been applied in other European countries with similar species compositions (e.g., Switzerland – Landolt and Sartori 2001; Czech Republic – Zahrádková et al. 2009), which enables comparison.

Since sampling frequency and collection methods changed considerably among sampling localities, we decided not to compare site-level mayfly diversity. However, we divided the studied altitudinal gradient into four 500 m-wide altitudinal belts (< 500 m, 501–1000 m, 1001–1500 m, and > 1501 m) and estimated the total number of mayfly species (the concept of gamma diversity according to Whittaker 1960) expected in each belt, which diminished the potential bias introduced by different sampling strategies. Since the number of localities varied widely among the belts (80–1528 localities), we standardised the sampling effort and estimated the expected total number of species at 80 localities in each belt using sample-based interpolation (Colwell et al. 2004).

Since mayflies are predominantly stream-dwelling insects, we also explored diversity patterns among streams of different orders. Each lotic site was classified according to Strahler’s system using the EU-Hydro River-Net dataset, a high-resolution pan-European hydrographic database (Gallaun et al. 2019). Again, sample-based interpolation was used to estimate the total number of species in each stream order, standardising the sampling effort to 100 streams. We restricted the analysis to localities with stream orders ranging from 1 to 6 because higher-order streams were sampled rarely.

The analysis was performed in R (R Development Core Team 2022) using the packages ggplot2 (Wickham 2016) and iNEXT (Chao et al. 2014).

## ﻿Results

### ﻿Checklist

**Figure 2.**
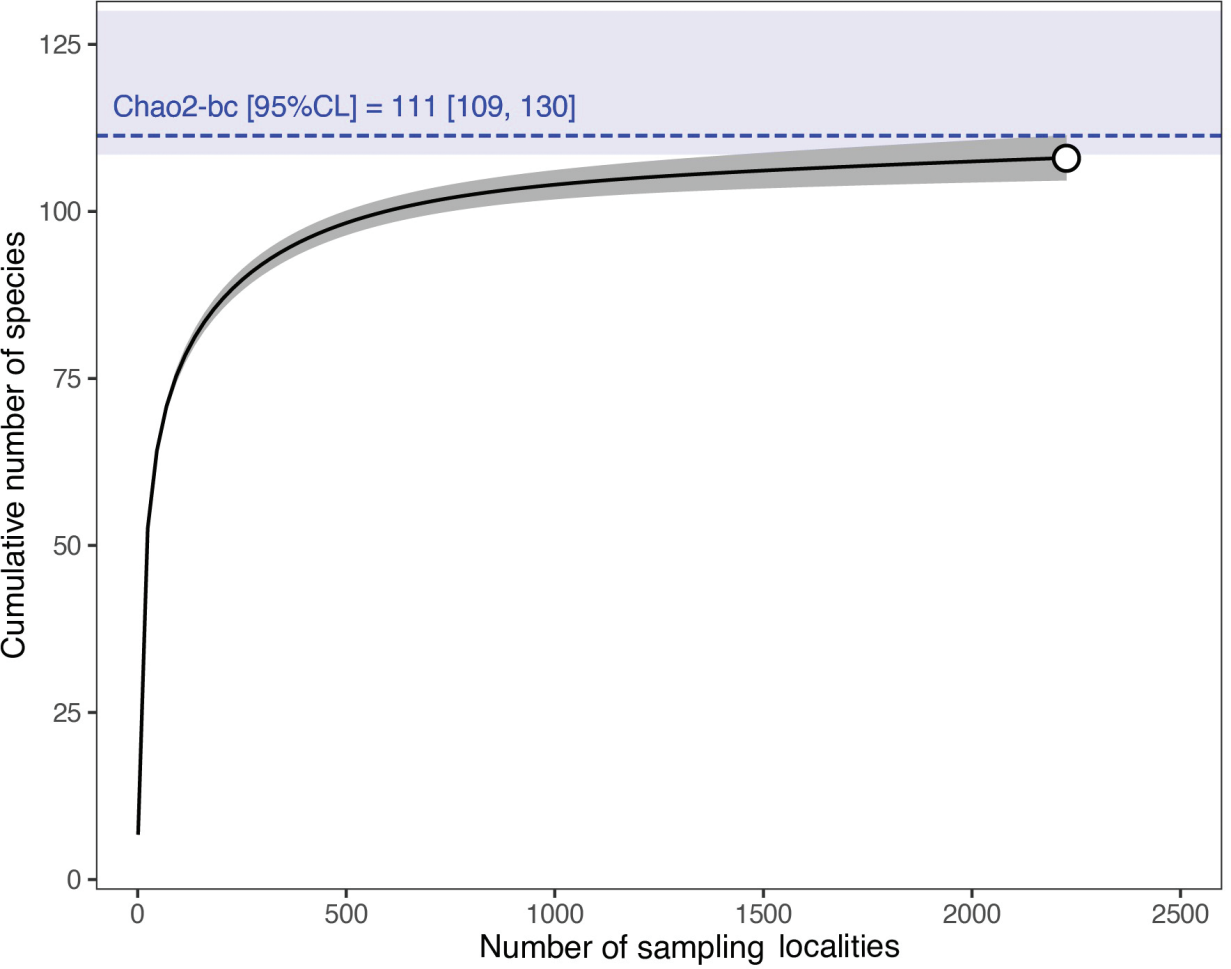
Sample-based rarefaction curve of mayfly species richness in Slovakia. The shaded area around the curve indicates the 95% confidence interval. The dashed line (± 95% confidence interval in blue) represents an estimate of the total number of species based on the bias-corrected Chao2 estimator (Chao2-bc).

Our extensive field survey resulted in the identification of 91 mayfly species. A literature review of all available publications related to mayflies revealed additional 40 taxa. Finally, the total number of species ever reported from the territory of Slovakia is 131. However, the detection of 15 species was evaluated as dubious, and seven observations were assigned “*species inquirenda*”. Hence, the current checklist of mayflies includes 109 species (Table 1). The delimitation of four species from the genus *Rhithrogena* is unclear, and therefore, we treated them as *Rhithrogenairidina* + *R.picteti* and *R.carpatoalpina* + *R.puytoraci*. *Ecdyonurusnigrescens* is reported with cf. (confer) due to ambiguous determining characters, which requires future comparison with the type material. More than 55% of the species belong to families Heptageniidae and Baetidae, nine families are monogeneric, and seven are monospecific. Finally, we report the following species richness for the detected families: Heptageniidae – 35, Baetidae – 27, Caenidae – 12, Leptophlebiidae – 9, Ephemerellidae – 5, Oligoneuridae, Ephemeridae and Siphlonuridae – 4, Ameletidae – 2, and Ametropodidae, Isonychiidae, Arthropleidae, Behningiidae, Palingeniidae, Polymitarcyidae and Potamanthidae – 1. The mayfly species inventory of Slovakia seems to be nearly complete as the accumulation curve reached an asymptote (Fig. 2). The expected total number of species calculated by the bias-corrected Chao2 estimator was 111, meaning that only two species are expected to remain undetected.

**Table 1.** Checklist of mayflies occurring in Slovakia and their species rarity (F1 to F5, see Material and Methods) according to Sartori and Landolt (1999), with the number of findings in brackets and the period of the last record according to the year of publication (++++ = ≤1990; +++ = 1991–2000; ++ = 2001–2010; + = 2011–2021). Excluded species are presented at the end of the checklist (*species inquirenda* and dubious findings). The species list is arranged by family, genus (subgenus), and species according to taxonomic nomenclature of Bauernfeind and Soldán (2012).

Taxa	Species rarity	Last record
**AMELETIDAE** Mc Cafferty, 1991		
***Ameletus*** Eaton, 1885		
*Ameletusinopinatus* Eaton, 1887	F4 (92)	+
***Metreletus*** Demoulin, 1951		
*Metreletusbalcanicus* (Ulmer, 1920)	F1 (8)	+
**SIPHLONURIDAE** Ulmer, 1920 (1888)		
**Siphlonurus (Siphlonurus)** Eaton, 1868		
Siphlonurus (Siphlonurus) aestivalis Eaton, 1903	F3 (43)	+
Siphlonurus (Siphlonurus) armatus Eaton, 1870	F2 (17)	+
Siphlonurus (Siphlonurus) lacustris Eaton, 1870	F2 (19)	+
**Siphlonurus (Siphlurella)** Say, 1824		
Siphlonurus (Siphlurella) alternatus (Say, 1824)	F1 (5)	++
**AMETROPODIDAE** Bengtsson, 1913		
***Ametropus*** Albarda, 1878		
*Ametropusfragilis* Albarda, 1878	F1 (4)	+
**BAETIDAE** Leach, 1815		
**Baetis (Acentrella)** Bengtsson, 1912		
Baetis (Acentrella) inexpectatus (Tshernova, 1928)	F1 (7)	++++
Baetis (Acentrella) sinaicus Bogoescu, 1931	F2 (12)	+
**Baetis (Baetis)** Leach, 1815		
Baetis (Baetis) alpinus (Pictet, 1843)	F5 (621)	+
Baetis (Baetis) buceratus Eaton, 1870	F5 (288)	+
Baetis (Baetis) fuscatus (Linnaeus, 1761)	F5 (453)	+
Baetis (Baetis) liebenauae Keffermüller, 1974	F3 (26)	+
Baetis (Baetis) lutheri Müller-Liebenau, 1960	F5 (349)	+
Baetis (Baetis) melanonyx (Pictet, 1843)	F5 (153)	+
Baetis (Baetis) pentaphlebodes Ujhelyi, 1966	F5 (136)	+
Baetis (Baetis) scambus Eeaton, 1870	F5 (287)	+
Baetis (Baetis) tracheatus Keffermüller & Machel, 1967	F1 (8)	+
Baetis (Baetis) vardarensis Ikonomov, 1962	F5 (221)	+
Baetis (Baetis) vernus Curtis, 1834	F5 (529)	+
**Baetis (Labiobaetis)** Novikova & Kluge, 1987		
Baetis (Labiobaetis) tricolor Tshernova, 1928	F2 (24)	+
**Baetis (Nigrobaetis)** Novikova & Kluge, 1987		
Baetis (Nigrobaetis) gracilis Bogoescu & Tabacaru, 1957	F1 (6)	++
Baetis (Nigrobaetis) muticus (Linnaeus, 1758)	F5 (506)	+
Baetis (Nigrobaetis) niger (Linnaeus, 1761)	F4 (54)	+
**Baetis (Rhodobaetis)** Jacob, 2003		
Baetis (Rhodobaetis) rhodani (Pictet, 1843)	F5 (1322)	+
**Baetopus (Raptobaetopus)** Müller-Liebenau, 1978		
Baetopus (Raptobaetopus) tenellus (Albarda, 1878)	F1 (5)	+
***Centroptilum*** Eaton, 1869		
*Centroptilumluteolum* (O. F. Müller, 1776)	F5 (135)	+
**Cloeon (Cloeon)** Leach, 1815		
Cloeon (Cloeon) dipterum (Linnaeus, 1761)	F5 (282)	+
**Cloeon (Similicloeon)** Kluge & Novikova, 1992		
Cloeon (Similicloeon) simile Eaton, 1870	F3 (32)	+
**Procloeon (Procloeon)** Bengtsson, 1915		
Procloeon (Procloeon) bifidum (Bengtsson, 1912)	F4 (78)	+
Procloeon (Procloeon) ornatum Tshernova, 1928	F1 (9)	++
**Procloeon (Pseudocentroptilum)** Bengtsson, 1915		
Procloeon (Pseudocentroptilum) macronyx Kluge & Novikova, 1992	F1 (7)	+
Procloeon (Pseudocentroptilum) pennulatum (Eaton, 1870)	F3 (49)	+
Procloeon (Pseudocentroptilum) pulchrum (Eaton, 1885)	F1 (1)	+
**ISONYCHIIDAE** Ulmer, 1914		
**Isonychia (Isonychia) Eaton, 1871**		
Isonychia (Isonychia) ignota (Walker, 1853)	F1 (8)	+
**OLIGONEURIDAE** Ulmer, 1914		
***Oligoneuriella* Ulmer, 1924**		
*Oligoneuriellapallida* (Hagen, 1855)	F1 (1)	++++
*Oligoneuriellapolonica* Mol, 1984	F1 (1)	++++
*Oligoneuriellarhenana* (Imhoff, 1852)	F4 (90)	+
***Oligoneurisca* Lestage, 1938**		
*Oligoneuriscaborysthenica* (Tshernova 1937)	F1 (1)	++++
**ARTHROPLEIDAE** Balthasar, 1937		
***Arthroplea* Bengtsson, 1908**		
*Arthropleacongener* Bengtsson, 1908	F1 (6)	+
**HEPTAGENIIDAE** Needham, 1901		
**Ecdyonurus (Ecdyonurus)** Eaton, 1871		
Ecdyonurus (Ecdyonurus) aurantiacus (Burmeister, 1839)	F4 (87)	+
Ecdyonurus (Ecdyonurus) dispar (Curtis, 1834)	F5 (132)	+
Ecdyonurus (Ecdyonurus) insignis (Eaton, 1870)	F3 (32)	+
Ecdyonurus (Ecdyonurus) macani Thomas & Sowa, 1970	F5 (138)	+
Ecdyonurus (Ecdyonurus) starmachi Sowa, 1971	F5 (249)	+
Ecdyonurus (Ecdyonurus) submontanus Landa, 1969	F5 (146)	+
Ecdyonurus (Ecdyonurus) torrentis Kimmins, 1942	F5 (212)	+
Ecdyonurus (Ecdyonurus) venosus (Fabricius, 1775)	F5 (263)	+
**Ecdyonurus (Helvetoraeticus)** Bauernfeind & Soldán, 2012		
Ecdyonurus (Helvetoraeticus) carpathicus Sowa, 1973	F4 (55)	+
Ecdyonurus (Helvetoraeticus) cf. nigrescens Klapálek, 1908	F1 (4)	+
Ecdyonurus (Helvetoraeticus) picteti (Meyer-Dür, 1864)	F2 (15)	+
Ecdyonurus (Helvetoraeticus) subalpinus Klapálek, 1907	F5 (168)	+
***Electrogena*** Zurwerra & Tomka, 1985		
*Electrogenaaffinis* (Eaton, 1883)	F2 (21)	+
*Electrogenalateralis* (Curtis, 1834)	F5 (129)	+
*Electrogenaquadrilineata* (Landa, 1969)	F2 (25)	+
*Electrogenaujhelyii* (Sowa, 1981)	F5 (158)	+
**Heptagenia (Dacnogenia)** Kluge, 1987		
Heptagenia (Dacnogenia) coerulans Rostock, 1878	F3 (31)	+
**Heptagenia (Heptagenia)** Walsh, 1863		
Heptagenia (Heptagenia) flava Rostock, 1878	F3 (141)	+
Heptagenia (Heptagenia) longicauda (Stephens, 1836)	F2 (15)	+
Heptagenia (Heptagenia) sulphurea (Müller, 1776)	F5 (166)	+
**Heptagenia (Kageronia)** Matsumura, 1931		
Heptagenia (Kageronia) fuscogrisea (Retzius, 1783)	F1 (6)	+
**Epeorus (Epeorus)** Eaton, 1881		
Epeorus (Epeorus) assimilis Eaton, 1885	F5 (504)	+
***Rhithrogena*** Eaton, 1881		
*Rhithrogenabeskidensis* Alba-Tercedor & Sowa, 1987	F5 (119)	+
*Rhithrogenacarpatoalpina*Klonowska et al. 1987 + *Rhithrogenapuytoraci* Sowa & Degrange, 1987	F5 (491)	+
*Rhithrogenacircumtatrica* Sowa & Soldán, 1986	F3 (34)	+
*Rhithrogenagermanica* Eaton, 1885	F4 (59)	+
*Rhithrogenagorganica* Klapálek, 1907	F1 (9)	++
*Rhithrogenahercynia* Landa, 1969	F3 (36)	+
*Rhithrogenairidina* (Kolenati, 1839) + *Rhithrogenapicteti* Sowa, 1971	F5 (463)	+
*Rhithrogenaloyolaea* Navás, 1922	F5 (107)	+
*Rhithrogenapodhalensis* Sowa & Soldán, 1986	F1 (5)	+
*Rhithrogenasavoiensis* Alba-Tercedor & Sowa, 1987	F3 (44)	+
*Rhithrogenasemicolorata* (Curtis, 1834)	F5 (608)	+
**LEPTOPHLEBIIDAE** Banks, 1900		
**Choroterpes (Choroterpes)** Eaton, 1881		
Choroterpes (Choroterpes) picteti (Eaton, 1871)	F1 (10)	+
***Habroleptoides*** Schönemund, 1929		
*Habroleptoidesconfusa* Sartori & Jacob, 1986	F5 (542)	+
***Habrophlebia*** Eaton, 1881		
*Habrophlebiafusca* (Curtis, 1834)	F4 (99)	+
*Habrophlebialauta* Eaton, 1884	F5 (306)	+
***Leptophlebia*** Westwood, 1840		
*Leptophlebiamarginata* (Linnaeus, 1767)	F1 (7)	+
*Leptophlebiavespertina* (Linnaeus, 1758)	F1 (8)	+
***Paraleptophlebia*** Lestage, 1917		
*Paraleptophlebiacincta* (Retzius, 1783)	F1 (10)	++
*Paraleptophlebiasubmarginata* (Stephens, 1836)	F5 (114)	+
*Paraleptophlebiawerneri* Ulmer, 1920	F2 (15)	+
**BEHNINGIIDAE** Motaş & Băcesco, 1937		
***Behningia*** Lestage, 1929		
*Behningiaulmeri* Lestage, 1929	F1 (1)	++++
**EPHEMERIDAE** Latreille, 1810		
**Ephemera (Ephemera)** Linnaeus, 1758		
Ephemera (Ephemera) danica Müller, 1764	F5 (481)	+
Ephemera (Ephemera) lineata Eaton, 1870	F4 (65)	+
Ephemera (Ephemera) vulgata Linnaeus, 1758	F4 (74)	+
**Ephemera (Sinephemera)** Kluge, 2004		
Ephemera (Sinephemera) glaucops Pictet, 1843	F1 (7)	++++
**PALINGENIIDAE** Albarda, 1888		
***Palingenia*** Burmeister, 1839		
*Palingenialongicauda* (Olivier, 1791)	F2 (13)	+
**POLYMITARCYIDAE** Banks, 1900		
***Ephoron*** Williamson, 1802		
*Ephoron virgo* (Olivier, 1791)	F3 (43)	+
**POTAMANTHIDAE** Albarda, 1888		
***Potamanthus*** Pictet, 1843		
*Potamanthusluteus* (Linnaeus, 1767)	F5 (215)	+
**EPHEMERELLIDAE** Klapálek, 1909		
***Ephemerella*** Walsh, 1862		
*Ephemerellaignita* (Poda, 1761)	F5 (576)	+
*Ephemerellamesoleuca* (Brauer, 1857)	F2 (16)	++
*Ephemerellamucronata* (Bengtsson, 1909)	F5 (468)	+
*Ephemerellanotata* Eaton, 1887	F4 (72)	+
***Torleya*** Lestage, 1917		
*Torleyamajor* (Klapálek, 1905)	F5 (211)	+
**CAENIDAE** Newman, 1853		
***Brachycercus*** Curtis, 1834		
*Brachycercuseuropaeus* Kluge, 1991	F1 (4)	++
*Brachycercusharrisellus* Curtis, 1834	F2 (14)	+
***Cercobrachys*** Soldán, 1986		
*Cercobrachysminutus* (Tshernova, 1952)	F1 (2)	+
***Caenis*** Stephens, 1835		
*Caenisbeskidensis* Sowa, 1973	F4 (92)	+
*Caenishoraria* (Linnaeus, 1758)	F5 (118)	+
*Caenislactea* (Burmeister, 1839)	F1 (8)	+
*Caenisluctuosa* (Burmeister, 1839)	F5 (301)	+
*Caenismacrura* Stephens, 1836	F5 (288)	+
*Caenispseudorivulorum* Keffermüller, 1960	F5 (119)	+
*Caenispusilla* Navás, 1913	F1 (2)	++
*Caenisrivulorum* Eaton, 1884	F3 (48)	+
*Caenisrobusta* Eaton, 1884	F4 (91)	+
**SPECIES INQUIRENDA**		
Baetis (Baetis) beskidensis Sowa, 1972	F1 (9)	+
Baetis (Rhodobaetis) gemellus Eaton, 1885	F2 (14)	+++
Cloeon (Cloeon) cognatum Stephens, 1836	F2 (21)	++
Cloeon (Cloeon) inscriptum Bengtsson, 1914	F2 (11)	+
Cloeon (Similicloeon) praetextum Bengtsson, 1914	F1 (6)	++
Procloeon (Pseudocentroptilum) nana (Bogoescu, 1951)	F1 (3)	+
*Rhithrogenazelinkai* Sowa & Soldán, 1984	F1 (4)	++
**DUBIOUS FINDINGS**		
Baetis (Baetis) macani Kimmins, 1957	F1 (1)	+
Baetis (Baetis) subalpinus Bengtsson, 1917	F2 (17)	++
Baetis (Nigrobaetis) digitatus Bengtsson, 1912	F1 (4)	++
*Oligoneuriellakeffermuellerae* Sowa, 1973	F1 (1)	+
Ecdyonurus (Helvetoraeticus) austriacus Kimmins, 1958	F1 (1)	+++
Ecdyonurus (Helvetoraeticus) epeorides Demoulin, 1955	F1 (2)	+++
Ecdyonurus (Helvetoraeticus) helveticus (Eaton, 1885)	F4 (70)	++
Ecdyonurus (Helvetoraeticus) zelleri (Eaton, 1885)	F2 (19)	++
*Ecdyonurusforcipula* (Pictet, 1843)	F4 (69)	+++
*Rhithrogenaalpestris* Eaton, 1885	F1 (1)	++++
*Rhithrogenadorieri* Sowa, 1971	F1 (2)	+++
*Rhithrogenahybrida* Eaton, 1885	F4 (65)	+
*Rhithrogenalandai* Sowa & Soldán, 1984	F1 (1)	++++
*Rhithrogenawolosatkae* Klonowska, 1987	F1 (3)	+++
*Palingeniafuliginosa* (Georgi, 1802)	F1 (8)	+

### ﻿Species distribution and diversity

**Figure 3.**
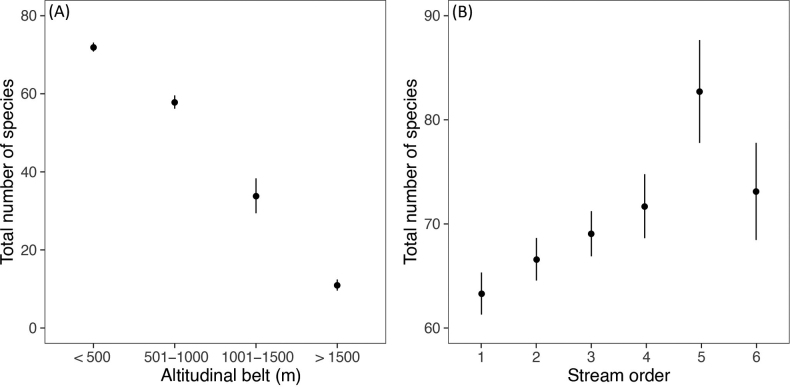
Total number of mayfly species (gamma diversity) according to **A** altitudinal belts and **B** stream orders. The estimated total number of species (black circle) is displayed with 95% confidence intervals (error bars).

*Baetisrhodani* was the most frequently occurring species, whereas *Behningiaulmeri*, *Oligoneuriellapolonica*, *O.pallida*, and *Oligoneuriscaborysthenica* were found at only a single location. Most of the mayfly species had optimal altitudinal distributions below 500 m, and only a few species were typical of the mountain areas (e.g., *Ameletusinopinatus*, *Rhithrogenaloyolaea*, *Rhithrogenahercynia*, and *Rhithrogenacircumtatrica*) (Suppl. material 2). The broadest amplitudes were recorded for *Baetisalpinus* (elevation range of 1982 m), *B.vernus* (1877 m), and *B.rhodani* (1871 m). Mayfly preferences for lower altitudes were also apparent in the gamma diversity patterns. The highest number of species was found at elevations below 500 m a.s.l. (72 spp.), while the gamma diversity decreases steeply towards higher altitudes (Fig. 3A). Considering the lotic species, mayfly gamma diversity showed a unimodal pattern along stream orders, with the highest number of species expected in streams of the fifth order (83 spp.) (Fig. 3B).

### ﻿Species rarity

Six species, which we considered extinct (refer to Discussion), are dated to a period before 1990, none between 1991 and 2000 and eight between 2001 and 2010. Only 95 species have been recorded since 2011. At the same time, we tried to confirm all species found before 2010 with our field research but without a positive result (Table 1). Applying the five-degree frequency scale to our data, we can classify 29 species as very rare (F1), 12 as rare (F2), 11 as uncommon (F3), 13 as common (F4) and 42 as widespread (F5) (Table 1, Suppl. material 3A). Moreover, species with very rare and rare frequencies usually occupy localities at lower altitudes (according to the median value) and in the lower altitudinal range, in contrast to those with very frequent distributions (Suppl. material 3B, C).

## ﻿Discussion

### ﻿Checklist

This study presents the first critically revised checklist of mayflies in Slovakia after two decades (Derka 2003a), listing 109 species from 33 genera and 16 families. This species list can be considered almost complete, as evidenced by the rarefaction analysis and nonparametric estimation.

In our list, six species from the family Baetidae and one from the Heptageniidae are marked as “*species inquirenda*”. Among those, the most frequently reported is *Baetisgemellus*. The first taxonomic ambiguities occurred when Steinmann (1907) described nymphs of *B.alpinus* as *B.gemellus*, and several authors followed this interpretation (e.g., Lestage 1918). However, the description of the nymphs does not exist, and male imagoes are very similar to *Baetisgadeai* (Thomas, 1999) or *B.rhodani* (Bauernfeind and Soldán 2012). Kimmins (1960) regarded *B.gemellus* sensu Steinman as a junior synonym of *B.alpinus* and *B.gemellus* sensu Eaton as identical to the closely related *B.rhodani*. Several authors followed this interpretation, including in reports from Slovakia (e.g., Landa 1969; Landa and Soldán 1989; Deván 1994). Therefore, all these findings of *B.gemellus* probably represent *B.rhodani*. Another species excluded from the checklist is *B.beskidensis* Sowa, 1972, reported by Deván (1992, 1993) and Mišíková-Elexová et al. (2010, 2015) from nine localities in our territory. The morphological separation, especially in the nymphal stage, from *B.fuscatus* or *B.scambus* is probably doubtful due to the minute details, mainly in the right mandible and labial palp (for a synopsis, see Bauernfeind and Soldán 2012).

Three species from the genus *Cloeon* (*C.cognatum*, *C.inscriptum*, *C.praetextum*) and one from *Procloeon* (*P.nana*) are also considered “*species inquirenda*”. Nymphs of *C.cognatum* and *C.incriptum* cannot be reliably separated from *C.dipterum*, and the differences in the imagoes are probably based on misidentification (Bauernfeind and Soldán 2012). Therefore, records of these species in Slovakia probably belong to *C.dipterum*. *Cloeonpraetextum* is very similar to *C.simile* in all stages, except for minor differences in egg chorion sculpture (Sowa 1980). However, such morphological features change considerably during development and have only weak diagnostic value (Bauernfeind and Soldán 2012). Accordingly, records of *C.praetextum* reported by Deván (1999, 2005) are considered conspecific with *C.simile*.

*Procloeonnana*, described initially as *Centroptilumnana* by Bogoescu (1951), was in the past placed in several genera (e.g., *Cloeon*; *Pseudocentroptiloides* Jacob, 1986) and probably represented the most problematic species in the category “*species inquirenda*”. The original description was elementary and based only on male and female imagoes. Subsequently, several authors published data on the occurrence of *Centroptilumnana* and a description of the nymph from different parts of Europe, which only led to other ambiguities in the description of this species (for a synopsis, see Głazaczow and Kłonowska-Olejnik 2009). However, Bauernfeind and Soldán (2012) suggest that *P.nana* might represent a senior subjective synonym of *Procloeonmacronyx*. In our checklist, three findings of *P.nana* were therefore considered synonymous with *P.macronyx*.

*Rhithrogenazelinkai* is the last species in the “*species inquirenda*” category. Descriptions of the subimago and imago of this species do not exist, and nymphs are closely related to *R.loyolaea* and *R.gorganica*. Finally, nymphs are not always identifiable without a doubt and are therefore considered “*species inquirenda*” (Bauernfeind and Soldán 2012).

The second taxon category excluded from the checklist represents 15 dubious species from four families (Baetidae, Heptageniidae, Oligoneuriidae, and Palingeniidae). Several species were identified in our territory by only one author, and most of them were identified in a single location (*Baetismacani*, *Ecdyonurusaustriacus*, *Rhithrogenaalpestris*, and *R.landai*) or the same stream/river (*R.dorieri* and *R.wolosatkae*), but subsequent investigations never confirmed their occurrence. Moreover, their distribution in Slovakia is very unlikely for several reasons. For example, the distribution of *Baetismacani* is restricted to northern Europe, with the southernmost limit in northern Germany and northeast Poland, where it is a typical lowland species (Bauernfeind and Soldán 2012). In our territory, its identification by Mišíková-Elexová et al. (2015) is therefore doubtful, and it was probably confused with the closely related and superficially similar *B.vernus*. The distribution of *Rhithrogenaalpestris* is mainly restricted to the Pyrenees and Alps, including mountain ranges in Slovenia (Bauernfeind and Soldán 2012). Nymphs prefer hypocrenal to epirhithral sections of brooks and small rivers at higher elevations, and their occurrence in the Danube River near Bratislava, as reported by Landa (1969), is highly improbable. *Rhithrogenalandai* is another species with a primary distribution in the Alps, including Slovenia and farther north in the Czech Republic and mostly in meta- and hyporhithral sections of streams (Bauernfeind and Soldán 2012). Accordingly, the record from a typical lowland river in our territory (Little Danube near Bratislava) reported by Landa and Soldán (1989) is doubtful. *R.dorieri* represents a West Alpine faunistic element, and the distribution of *R.wolosatkae* is probably restricted to the Pyrenees (Bauernfeind and Soldán 2012); therefore, we considered their findings at single localities (Deván 1995a; Krno et al. 1996) as dubious. *Ecdyonurusepeorides* was reported by Deván (1993, 1999) from two localities, but the occurrence of this species is also improbable since the species is likely restricted to the southeastern Balkans (Bauernfeind and Soldán 2012). *Oligoneuriellapallida* was reported by Mišíková-Elexová et al. (2015) in a tributary of the Slaná River with a typical occurrence of *O.rhenana* (Mišíková-Elexová et al. 2015) and according to Bauernfeind and Soldán (2012), nymphs of *O.pallida* never coexist with *O.rhenana*. In contrast, they prefer the metapotamal sections of larger rivers at lower altitudes and never occur in streams. Therefore, we consider its records dubious and probably based on misidentification. Finally, *O.pallida* was recorded from Slovakia only once and in a single location by Soldán (1978). We examined the material of *Baetisdigitatus* and *Oligoneuriellakeffermuellerae* recorded by Mišíková-Elexová et al. (2010, 2021) from all known localities in Slovakia and stored at the Department of Ecology, Commenius University, Bratislava, Slovakia. After the taxonomic revision, we considered these findings to be dubious due to misidentifications. Finally, the nymphs of *O.keffermuellarae* corresponded to *O.rhenana* and *Baetisdigitatus* to *B.niger*. Moreover, Mišíková-Elexová et al. (2021) reported *Oligoneuriellakeffermullerae* in the Poprad River and its very close tributary, both typical of the occurrence of *O.rhenana*, which was also confirmed by our records. Furthermore, *O.keffermullerae* never co-occurs with *O.rhenana* (Bauernfeind and Soldán 2012).

The most frequently reported dubious specie were *Ecdyonurusforcipula*, *E.helveticus*, *E.zelleri*, *Rhithrogenahybrida*, and *Baetissubalpinus*. The occurrence of *Ecdyonurusforcipula* sensu Pictet (1843–1845) in our territory corresponds to several taxa from the *E.venosus* species group. Therefore, we consider the records of this species dubious. The records of *E.helveticus* and *E.zelleri* were reported mostly by Deván (e.g., 1993; 1995a, b; 1996a, b; 1999), Derka (1995), Krno et al. (1996), and Krno (1997a, b). However, these records are probably based on misidentification since the distribution of these species is primarily limited to the Alps (Bauernfeind and Soldán 2012). Moreover, nymphs of *E.helveticus* are very similar to *E.picteti*, and findings in Slovakia may thus correspond to the latter species. Another erroneously determined species is *Rhithrogenahybrida*, whose distribution is probably restricted to the Alps (Black Forest, Germany, Bauernfeind and Soldán 2012). Records from Slovakia probably correspond to other representatives of the *R.hybrida* species group, e.g., *R.podhalensis* or *R.circumtatrica* (Bauernfeind & Soldán, 2012). The last species relatively often reported as dubious was *Baetissubalpinus* (e.g., Krno 1991, 1997a; Derka 1995, 2003c; Deván 1999, 2000). However, findings from central and southern Europe are questionable and probably also based on misidentification because nymphs are superficially similar to *B.vernus* in all stages (Bauernfeind and Soldán 2012). Finally, the latest morphological and molecular analyses confirmed the dubious taxonomical status of *Palingeniafuliginosa* in southeastern Europe (Manko et al. 2023), including in reports from Slovakia (Landa 1969; Soldán 1981a; Landa and Soldán 1989; Mišíková-Elexová et al. 2015).

### ﻿Species distribution and diversity

We have shown that most mayfly species occurring in Slovakia prefer habitats at lower elevations (< 500 m), and overall mayfly diversity continually decreases from lowland to mountain areas. This agrees with the general observations that high mountain habitats are relatively poor in mayfly species richness (e.g., Landolt and Sartori 2001), and the number of mayfly species decreases with increasing altitude (Brittain and Sartori 2009). According to the habitat classification of running waters (Illies 1953; Illies and Botosaneanu 1963), the highest aquatic species richness occurs in lotic-erosional habitats or rhithral sections. The taxa richness of mayflies in Europe is particularly high in meta- and hyporhithral sections in the colline or lower mountain zones (Landolt and Sartori 2001; Bauernfeind and Soldán 2012). These sections correspond to most habitats of fifth-order streams in Slovakia, the stream order with the highest recorded mayfly gamma diversity. Regarding mayflies, the potamal sections or lotic-depositional habitats are less species-rich (Bauernfeind and Soldán 2012). Such habitats correspond to higher stream orders (≥ 6^th^ stream order in Slovakia), with low species richness, but are usually occupied by several ecological specialists with a very low frequency of occurrence (see below) and being mostly restricted to these river sections (Landolt and Sartori 2001). In contrast, Bauernfeind and Moog (2000) showed the highest species and family richness within ecologically intact to moderately disturbed sampling localities in the potamal sections due to the high diversity of microhabitats. However, lowland rivers are typically exposed to extensive chemical pollution and suffer from the loss of habitat heterogeneity, current variability, and various substrate types needed for mayfly specialists (Zedková et al. 2015; Vilenica et al. 2020, 2022). Such negative impacts significantly affect the biodiversity of entire communities and cause the decline of many rare specialists, which are replaced by more tolerant species (Bauernfeind and Moog 2000; Rumschlag et al. 2023).

### ﻿Species rarity

According to their occurrence frequency, 29 species (26%) were evaluated as very rare. These species usually occupied localities at lower altitudes. According to the generally accepted classification of Baillie and Groombridge (1996), a species is considered extinct if its latest record is older than 30 years. Here, we list six species that meet this criterion: *Behningiaulmeri*, *Oligoneuriellapolonica*, *O.pallida*, *Oligoneuriscaborysthenica*, *Baetisinexpectatus*, and *Ephemeraglaucops*. *Behningiaulmeri*, *Oligoneuriellapolonica*, *O.pallida*, and *Oligoneuriscaborysthenica* were last reported by Landa and Soldán (1989) and Soldán (1978) from two sampling localities in the southeast part of Slovakia. These species prefer epi- and metapotamal sections of large rivers, where their nymphs inhabit coarse shifting sand (*Behningiaulmeri*, *Oligoneuriellapolonica*, *Oligoneuriscaborysthenica*) or stony bottom (*Oligoneuriellapallida*), which probably determines their occurrence (Bauernfeind and Moog 2000; Bauernfeind and Soldán 2012). *Baetisinexpectatus* and *Ephemeraglaucops* were reported from seven localities having thus scarce distributions. In Slovakia, *E.glaucops* was recorded in large lowland rivers (Landa and Soldán 1989) and one natural lake of volcanic origin with oligotrophic to mesotrophic conditions (Gajdúšek and Kubíček 1970). Nymphs were also found in different artificial waterbodies in central Europe (Jacob et al. 1975; Studemann et al. 1992; Sroka et al. 2022). The biology of *Baetisinexpectatus* is poorly known (Bauernfeind and Soldán 2012). In Slovakia, nymphs were recorded in medium-sized lowland rivers and always in habitats with maximum current velocity and stony substrata (Soldán 1981b; Landa and Soldán 1989). The fauna of the large lowland rivers and their floodplains has undergone the most significant changes in species composition caused mainly by human activities (Deván 2001; Soldán et al. 2017), which probably led to the extinction of the five species mentioned above in Slovakia. Recent reports from Ukraine confirmed the importance of lowland rivers for rare mayfly species (Martynov 2018, 2020). In some cases, it is plausible to expect the rediscovery of some rare species, as evidenced by our finding of *Cercobrachysminutus* in 2021, more than 40 years after the last record (Soldán 1978).

Among other very rare species, we found species that inhabit lentic habitats often overlooked during hydrobiological research and routine biomonitoring in Slovakia (Mišíková-Elexová et al. 2010, 2015, 2021) or that occur at the edge of their geographic distribution (Soldán et al. 1998). For example, *Leptophlebiavespertina* and *L.marginata* inhabit slow-flowing and slightly acidic streams, rivers, and, more frequently, lakes (Bauernfeind and Soldán 2012). In Slovakia, these species were found mainly in lentic habitats such as subalpine dystrophic lakes in the High Tatras (e.g., Landa and Soldán 1989; Krno 1991) and water reservoirs (Mišíková-Elexová et al. 2015). Species preferring stagnant waters include *Siphlonurusalternatus*, *Procloeonpulchrum*, and *Caenislactea* (Bauernfeind and Soldán 2012). *Siphlonurusalternatus* predominantly inhabits pools with submerged vegetation, isolated backwaters in the inundation areas of larger lowland rivers, oligotrophic ponds, artificial impoundments, and montane glacial lakes in Central Europe (Landa and Soldán 1989; Soldán et al. 1998; Bauernfeind and Soldán 2012).

*Metreletusbalcanicus* and *Arthropleacongener* are among taxa inhabiting rather specific and probably overlooked habitat type. *Metreletusbalcanicus* usually occurs in periodic slow-flowing streams with muddy and clay bottoms with or without aquatic vegetation (Russev and Vidinova 1994; Bauernfeind and Soldán 2012; Martynov 2018). *Arthropleacongener* was recently confirmed in two neighbouring localities in southwest Slovakia, while a relatively large population was recorded only in the shallow temporally inundated wetland in an alder forest (Macko and Derka 2021). *Ametropusfragilis*, *Brachycercuseuropaeus*, and *Heptageniafuscogrisea* are other “very rare” species that currently occur in only a few relatively preserved lowland rivers of southeastern Slovakia (e.g., Mišíková-Elexová et al. 2010, 2015, 2021) and/or the Ipeľ (Ipoly) River (Kovács et al. 2002; Macko and Derka 2021).

In contrast, the frequency of *Rhithrogenagorganica* was also evaluated as “very rare”, but this species is widely distributed in the Ukrainian Carpathians (e.g., Godunko 2000). Localities mostly represented by hypocrenal brooks in the beech forest situated in the northeastern part of Slovakia, the Nízke Beskydy Mountains (e.g., Novikmec et al. 2007) probably represent the westernmost limit of the distribution of this species endemic to the eastern Carpathians (Bauernfeind and Soldán 2012). *Caenispusilla* represents a Mediterranean faunistic element with a northern area extension (Malzacher 1986) or holomediterranean distribution (Haybach and Jacob 2010). Nymphs predominantly inhabit hyporhithral and epipotamal sections of rivers, especially with stony bottoms in Central Europe (Bauernfeind and Soldán 2012), as was confirmed in two known localities in Slovakia (Mišíková-Elexová et al. 2010). At the same time, they also probably represent the easternmost and only known localities in the western Carpathians (Derka 2005a). *Baetopustenellus* is a Transpalaearctic species with usually very low densities at sampling localities (Derka 2005b), predominantly in the epi- and metapotamal sections of large lowland rivers (Bauernfeind and Soldán 2012). In the 20^th^ century, *B.tenellus* was recorded in our territory at only a single locality, exceptionally representing the hyporhithral section of River Ulička (Landa and Soldán 1989), and at the beginning of the 21^st^ century, it was also discovered in the metapotamal section of the Morava River (Derka 2005b, Mišíková-Elexová et al. 2010). Our current findings represent three additional occurrence localities of this species and typical epi- and metapotamal sections of our large lowland rivers. The occurrence of *Isonychiaignota* in Slovakia remains rare and findings are irregular. This species was recently confirmed at four hyporhitral localities (Mišíková-Elexová et al. 2010, 2015). However, despite our repeated locality visits since 2019, we have not recorded this species.

## ﻿Conclusions

We present the first comprehensive checklist of mayflies in Slovakia based on century-long research, containing 109 species. Due to the high spatial heterogeneity of the region and the robustness of our dataset covering more than 2200 sampling localities, we believe that fundamental aspects of mayfly diversity revealed in our study can be generalised beyond the western Carpathians and Pannonia. The highest number of species was found at elevations below 500 m a.s.l. and decreases towards higher altitudes. Regarding stream longitudinal zonation, gamma diversity showed a unimodal pattern, with the highest number of species occurring in streams of the fifth order. Rare species mostly occurred in lower altitudes. Moreover, all six species are considered extinct in Slovakia, dwelling in lowland rivers. Owing to the high mayfly diversity and the occurrence of many rare species, lowland rivers and their floodplains deserve high priority for the conservation of mayflies in Central Europe.

## ﻿Additional information

### Author ORCIDs

Patrik Macko https://orcid.org/0009-0008-0714-7490

Tomáš Derka https://orcid.org/0000-0002-9304-9668

Michaela Šamulková https://orcid.org/0009-0000-9599-5255

Milan Novikmec https://orcid.org/0000-0002-5192-4575

Marek Svitok https://orcid.org/0000-0003-2710-8102

### Data availability

All of the data that support the findings of this study are available in the main text or Supplementary Information.

## Supplementary materials

Supplementary material 1 Details of the collection data of mayflies in Slovakia from 1905 to 2021 Data type xlsx https://binary.pensoft.net/file/927786This dataset is made available under the Open Database License (http://opendatacommons.org/licenses/odbl/1.0/). The Open Database License (ODbL) is a license agreement intended to allow users to freely share, modify, and use this Dataset while maintaining this same freedom for others, provided that the original source and author(s) are credited.Patrik Macko, Tomáš Derka, Michaela Šamulková, Milan Novikmec, Marek Svitok

Supplementary material 2 Altitudinal distribution of mayfly species in Slovakia Data type jpg Explanation note Species are ordered according to the position of their altitudinal optima (median of the distribution). The number of occurrence localities is given in italics for each species. Note that *R.iridina* and *R.carpatoalpina* also represent *R.picteti* and *R.puytoraci*, respectively. https://binary.pensoft.net/file/927787This dataset is made available under the Open Database License (http://opendatacommons.org/licenses/odbl/1.0/). The Open Database License (ODbL) is a license agreement intended to allow users to freely share, modify, and use this Dataset while maintaining this same freedom for others, provided that the original source and author(s) are credited.Patrik Macko, Tomáš Derka, Michaela Šamulková, Milan Novikmec, Marek Svitok

Supplementary material 3 Number of mayfly species in five frequency classes, and their altitudinal and geographical distributions Data type xlsx Explanation note **A** Number of mayfly species in five frequency classes (F1 to F5, see Materials and methods) defined by Sartori and Landolt (1999) and their **B** altitudinal and **C** geographical distributions. https://binary.pensoft.net/file/927788This dataset is made available under the Open Database License (http://opendatacommons.org/licenses/odbl/1.0/). The Open Database License (ODbL) is a license agreement intended to allow users to freely share, modify, and use this Dataset while maintaining this same freedom for others, provided that the original source and author(s) are credited.Patrik Macko, Tomáš Derka, Michaela Šamulková, Milan Novikmec, Marek Svitok
