# Supplementary figures and images for: ﻿Checklist, distribution, diversity, and rarity of mayflies (Ephemeroptera) in Slovakia
Source: Zookeys. 2023 Oct 27;1183:39–64. doi: 10.3897/zookeys.1183.109819 (PMC10836656; doi:10.3897/zookeys.1183.109819)

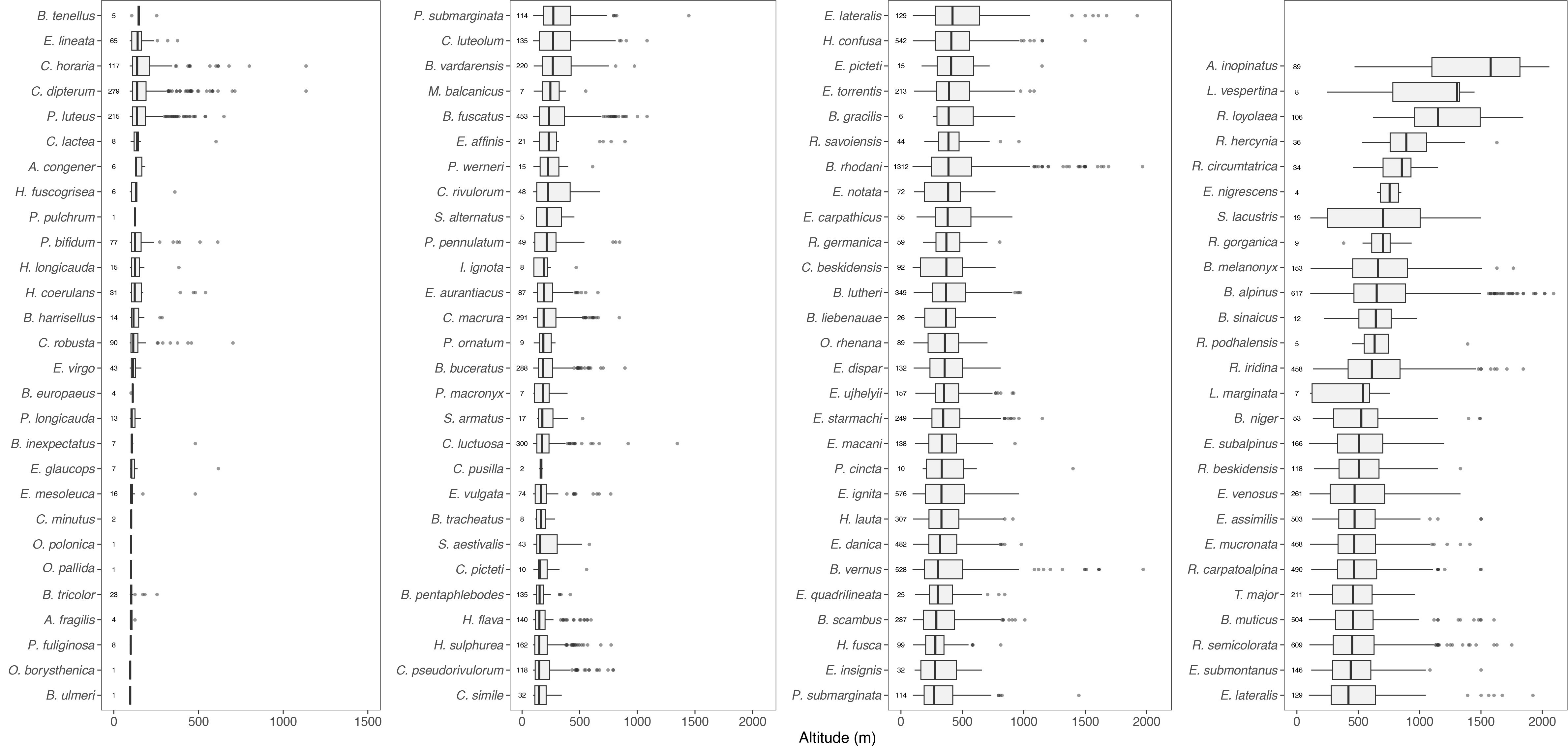

Supplement: Supplementary material 2 — Altitudinal distribution of mayfly species in Slovakia [file zookeys-1183-039_article-109819__-s002.jpg]
